# Intracerebral hemorrhage after external ventricular drain placement: an evaluation of risk factors for post-procedural hemorrhagic complications

**DOI:** 10.1186/s12883-018-1030-7

**Published:** 2018-03-07

**Authors:** A. Shaun Rowe, Derrick R. Rinehart, Stephanie Lezatte, J. Russell Langdon

**Affiliations:** 1 College of Pharmacy, Department of Clinical Pharmacy and Translational Science, The University of Tennessee Health Science Center, 1924 Alcoa Highway, Box 117, Knoxville, TN 37920 USA; 2 Department of Pharmacy, Parkwest Medical Center, Knoxville, TN USA; 3 The University of Tennessee Medical Center, University Neurocritical Care, Knoxville, TN USA; 4 Department of Anesthesiology, The University of Tennessee Graduate School of Medicine, Knoxville, TN USA

**Keywords:** Ventriculostomy, Cerebral hemorrhage, Risk factors, Retrospective studies, External ventricular drain, Prevention and control

## Abstract

**Background:**

The objective of this study was to evaluate and identify the risk factors for developing a new or enlarged intracranial hemorrhage (ICH) after the placement of an external ventricular drain.

**Methods:**

A single center, nested case-control study of individuals who received an external ventricular drain from June 1, 2011 to June 30, 2014 was conducted at a large academic medical center. A bivariate analysis was conducted to compare those individuals who experienced a post-procedural intracranial hemorrhage to those who did not experience a new bleed. The variables identified as having a *p*-value less than 0.15 in the bivariate analysis were then evaluated using a multivariate logistic regression model.

**Results:**

Twenty-seven of the eighty-one study participants experienced a new or enlarged intracranial hemorrhage after the placement of an external ventricular drain. Of these twenty-seven patients, 6 individuals received an antiplatelet within ninety-six hours of external ventricular drain placement (*p* = 0.024). The multivariate logistic regression model identified antiplatelet use within 96 h of external ventricular drain insertion as an independent risk factor for post-EVD ICH (OR 13.1; 95% CI 1.95–88.6; *p* = 0.008).

**Conclusion:**

Compared to those study participants who did not receive an antiplatelet within 96 h of external ventricular drain placement, those participants who did receive an antiplatelet were 13.1 times more likely to exhibit a new or enlarged intracranial hemorrhage.

## Background

External ventricular drains (EVD) are one of the most commonly performed neurosurgical procedures. They are used to provide real time intracranial pressure monitoring and treatment of elevated intracranial pressure [1, 2]. EVDs are often employed during the management of disease states such as traumatic brain injury, spontaneous intracranial hemorrhage (ICH), hydrocephalus, ventriculitis, and meningitis [1]. Though considered relatively safe, complications such as hemorrhage, infection, and malposition do occur [2].

The rate of EVD-associated hemorrhage ranges from 18 to 41%, and recent articles have suggested possible risk factors associated with hemorrhage. These risk factors include age, decreased platelets on admission, and increased number of EVD attempts [3–5]. In addition, a recent consensus statement released by the Neurocritical Care Society provides a “good practice statement” suggesting that the reversal of anticoagulants should occur prior to insertion of an EVD except in dire circumstances, and provides a conditional recommendation based on low quality of evidence that patients with an EVD and at high risk of venous thromboembolism (VTE) should receive pharmacologic VTE prophylaxis once intracranial hemorrhage is stable or absent [2]. As suggested by the level of this recommendation, literature concerning the effects of perioperative use of antiplatelet and anticoagulant agents in patients with an EVD is limited.

Because of the paucity of data concerning the use of VTE prophylaxis and antiplatelet medications in patients with an EVD, we sought to evaluate these and other potential risk factors for intracerebral hemorrhage following placement of an EVD.

## Methods

This was a nested case-control study designed to evaluate the risk factors associated with new or enlarged intracranial hemorrhage in patients with EVDs. The study was approved by The University of Tennessee Graduate School of Medicine Institutional Review Board committee. All patients included in this study were 18 years of age or older who had an EVD placed between June 1, 2011 and June 30, 2014. Those individuals without a pre-procedural and/or a post-procedural cranial computed tomography (CT) scan were excluded from the study.

Clinical and radiographic data including baseline demographics, EVD indication, coagulation parameters, anticoagulant use, antiplatelet use, Glasgow Coma Score (GCS), and presence of new or enlarged intracranial hemorrhage were collected through a retrospective review of the electronic medical record. If the cranial CT after EVD placement demonstrated a new or enlarged catheter-related bleed, the individual was grouped into a cohort as having evidence of a post-EVD ICH. All head CT scans with any evidence of a new or enlarged bleed were reviewed and quantified by a medical professional. Hemorrhage was specifically defined as a new or enlarged ICH on the ipsilateral side of EVD insertion or catheter tract hemorrhage of greater than 5 ml in volume or greater than 5 mm in any diameter as seen on CT scan after the placement of the EVD. If the bleed did not meet this predefined threshold, the individual was then removed from the EVD-related bleed cohort and grouped with those who did not experience a hemorrhagic complication.

The primary endpoint of this study was the identification of potential risk factors for new or enlarged intracranial hemorrhage after the placement of an EVD. Secondary endpoints included in-hospital mortality, hospital length of stay, and EVD duration in those with new or enlarged ICH and those without new or enlarged ICH.

Statistical analyses were conducted with SAS (SAS Institute, Cary, NC, Version 9.4 TS1M4). Continuous data were evaluated for normality using Shapiro-Wilk or Kolmogorov-Smirnov tests and visual evaluation of histograms. A bivariate analysis was conducted to compare participants considered to have a new or enlarged ICH after EVD placement to those grouped as having no evidence of new ICH after EVD placement. Normally distributed continuous variables were tested for statistical significance using a Student’s T-test and the non-normal continuous variables were compared with a Mann-Whitney U test. Nominal data was compared with a Chi-squared test or Fisher’s exact test, as appropriate. To identify the independent risk factors for EVD-associated hemorrhage, the variables with a *p* < 0.15, in which all data points were recorded, were evaluated using a multivariate logistic regression model.

## Results

Of the 126 patients evaluated for study inclusion, 81 patients were included. The remaining 45 patients were excluded because of age being less than 18 or the lack of both a pre- and post-procedural head CT scan. Of the 81 patients studied, 27 (33.3%) had evidence of a new or enlarged ICH after the placement of an EVD. Full baseline demographics and classification of new or enlarged hemorrhages can be seen in Tables 1 and 2 respectively. When the groups were compared, there were no statistically significant differences with respect to age, EVD indication, history of hypertension, presence of preadmission anticoagulant use, or presence of preadmission antiplatelet use.

**Table 1:** Baseline Characteristics of Patients

Parameter	No Evidence of New/Enlarged ICH (*n* = 54)	Evidence of New/Enlarged ICH (*n* = 27)	*p*-value
Age (years), mean ± SD ^a^	50.4 ± 20.1	57.8 ± 17.7	0.109
Male, *n* (%) ^b^	25 (46.3)	14 (51.9)	0.637
Caucasian, *n* (%) ^b^	47 (87)	24 (88.9)	0.617
Indication for EVD, *n* (%) ^b^			0.261
Head Trauma	16 (29.6)	6 (22.2)	
Non-traumatic ICH	21 (38.9)	18 (66.7)	
Hydrocephalus	10 (18.5)	2 (7.4)	
Meningitis/Ventriculitis	1 (1.9)	0 (0)	
Cerebral Edema	1 (1.9)	0 (0)	
Other	5 (9.3)	1 (3.7)	
History of HTN, *n* (%) ^b^	30 (55.6)	18 (66.7)	0.337
History of Tobacco, *n* (%) ^b^	16 (29.6)	10 (37)	0.501
Known Cocaine use, *n* (%) ^c^	2 (3.7%)	0 (0)	0.5500
Pre-admission Anticoagulation, *n* (%) ^c^	3 (5.6)	3 (11.1)	0.3949
Pre-admission Antiplatelet Therapy, *n* (%) ^b^	9 (16.7)	8 (29.6)	0.177
Intracerebral Hemorrhage present on admission, *n* (%) ^b^	45 (83.3%)	24 (88.9%)	0.5070

^a^Independent samples t-test

^b^Chi Square

^c^Fisher’s Exact

**Table 2:** Classification of new hemorrhage after EVD Insertion

Bleed Classification	Patients with a new hemorrhage after EVD Insertion, *n* (%)
Enlargement of Preexisting (pre-EVD) ICH by ≥5 mL in volume or ≥5 mm in largest diameter	11 (40.74%)
New Catheter Tract Hemorrhage ≥5 mL in volume or ≥5 mm in largest diameter	10 (37.04%)
Spontaneous New ICH ≥ 5 mL in volume or ≥5 mm in largest diameter	6 (22.22%)

Among the 27 patients who suffered a post-EVD ICH, only 1 (3.7%) received an anticoagulant and only 1 (3.7%) received an antiplatelet in the time from admission to time of EVD insertion. Overall, no statistically significant difference was found between patients who experienced a post-EVD ICH and those who did not with regard to preprocedural GCS, International Normalized Ratio (INR), and antithrombotic use (Table 3).

**Table 3:** Preprocedural Analysis

Parameter	No Evidence of New/Enlarged ICH (*n* = 54)	Evidence of New/Enlarged ICH (*n* = 27)	*p*-value
Admission GCS, (Median ± IQR) ^a^	10 ± 8.3	7 ± 9	0.1964
GCS (highest) 24 h prior to EVD, (Median ± IQR) ^a^	11.5 ± 9	8 ± 7	0.2827
GCS (lowest) 24 h prior to EVD, (Median ± IQR) ^a^			0.3437
INR (highest) from admission to EVD, (Median ± IQR) (*n* = 76) ^a^	1.1 ± 0.2	1.1 ± 0.2	0.3437
aPTT (highest) from admission to EVD, (Median ± IQR) ^a^	25.9 ± 4.5	27.3 ± 5.6	0.017
Preprocedural Anticoagulation, *n* (%) ^b^	4 (7.4)	1 (3.7)	0.66
Preprocedural Antiplatelet, *n* (%) ^b^	2 (3.7)	1 (3.7)	1.0

^a^Mann-Whitney U

^b^Chi Square

As it relates to our primary outcome of identifying risk factors for new or enlarged ICH after placement of an EVD, several statistically significant differences were identified in the post-procedural period (Table 4). As it relates to postoperative anticoagulant use, less patients in the post-EVD ICH group received an anticoagulant within 96 h of EVD insertion (14 [25.9%] vs. 2 [7.4%]; *p* = 0.048). Conversely, more patients in the post-EVD ICH group received an antiplatelet within 96 h of EVD insertion (6 [22.2%] vs. 3 [5.6%]; *p* = 0.0539). The type of antiplatelet agent received after EVD insertion can be seen in Table 5. Additionally, the average GCS on post-op day 3 and 4 was significantly lower in the group with post-EVD ICH (3 ± 6.8 and 4 ± 5.3) compared to the group without post-EVD ICH (8 ± 10 and 9 ± 10).

**Table 4:** Post-ventriculostomy Analysis

Parameter	No Evidence of New/Enlarged ICH (*n* = 54)	Evidence of New/Enlarged ICH (*n* = 27)	*p*-value
EVD placed by a trainee or mid-level practitioner, *n* (%) ^a^	19 (35.19)	8 (29.63%)	0.6171
Placement Attempts, (Median ± IQR) (*n* = 69) ^b^	1 (0)	1 (0)	0.8831
Anticoagulation within 96 h of EVD, *n* (%) ^a^	14 (25.9)	2 (7.4)	0.048
Prophylactic anticoagulation, *n* (%) ^a, d^	11 (20.4)	2 (7.4)	> 0.999
Antiplatelet within 96 h of EVD, *n* (%) ^c^	3 (5.6)	6 (22.2)	0.0539
GCS (lowest) POD 1, (Median ± IQR) ^b^	6 ± 6	6 ± 6	0.17
GCS (lowest) POD 2, (Median ± IQR) (*n* = 80) ^b^	6.5 ± 7.8	4.5 ± 6.5	0.0624
GCS (lowest) POD 3, (Median ± IQR) (*n* = 76) ^b^	8 ± 10	3 ± 6.8	0.0202
GCS (lowest) POD 4, (Median ± IQR) (*n* = 72) ^b^	9 ± 10	4 ± 5.3	0.0383

^a^Chi Square

^b^Mann-Whitney U

^c^Fisher’s Exact

^d^Inferential test was performed on in those patients who received anticoagulation

**Table 5:** Type of post-procedural anticoagulant and antiplatelet administered

	No Evidence of New/Enlarged ICH	Evidence of New/Enlarged ICH	*p*-value
Anticoagulant (*n* = 16)	*n* = 14	*n* = 2	> 0.999
Heparin	11 (78.57)	2 (100%)	
Enoxaparin	3 (21.43)	0 (0)	
Antiplatelet (*n* = 9)	*n* = 3	*n* = 6	0.6873
Aspirin	2 (66.67)	4 (66.67)	
Clopidogrel	1 (33.3)	1 (16.67)	
Prasugrel	0 (0)	1 (16.67)	

The variables that met the criteria for inclusion in the multivariate logistic regression model were postoperative anticoagulant use within 96 h of EVD insertion, postoperative antiplatelet use within 96 h of EVD insertion, lowest recorded GCS on postoperative day 3, and lowest recorded GCS on postoperative day 4. The logistics regression model only included those statistically significant variables for which 100% of the data points were available for collection. Thus, only 72 patients were included in the logistic regression model. In the final multivariate model, only the use of an antiplatelet agent within 96 h of EVD placement was a risk factor for post-EVD ICH (OR 13.1, 95% CI 1.95–88.6; *p* = 0.008). The full logistic regression model can be seen in Table 6.

**Table 6:** Multivariate Logistic Regression Predicting for Hematoma Expansion, *N* = 72

Variable	Wald Chi Square (df = 1)	*p*-value	Odds Ratio	95% CI
GCS (lowest) POD 3	0.5907	0.925	1.022	0.65–1.6
GCS (lowest) POD4	0.0088	0.442	0.837	0.53–1.3
Postoperative Anticoagulation	2.7454	0.098	0.193	0.03–1.4
Postoperative Antiplatelet	6.9952	0.008	13.14	1.95–88.6

Hosmer and Lemeshow Goodness-of-Fit (*p* = 0.5545)

As it relates to our secondary outcomes of mortality, a higher proportion of those patients who experienced a new or enlarged ICH after EVD placement died while in the hospital (16 [29.63%] vs. 17 [62.96%]; *p* = 0.0040). However, having a new or enlarged ICH was not associated with a longer hospital length of stay (18 days ±14 days vs. 15 ± 18; *p* = 0.1486). Nor was having a new or enlarged ICH after EVD placement related a longer EVD duration (8 ± 7 days vs. 7 days ±8 days; *p* = 0.8149).

## Discussion

The insertion of an EVD is a commonly performed neurosurgical procedure that is associated with a recognized risk of ICH. The reported rates of secondary hemorrhage after the placement of an EVD vary significantly with documented rates being as high as 41% [3]. Because of this significant rate of post-EVD ICH, the present study was designed to investigate the risk factors for post-EVD ICH. In our sample of patients, the prevalence of post-EVD ICH was 33.3%. This rate is consistent with the most recent studies that have evaluated EVD-related hemorrhagic complications.

Although several small studies have evaluated whether certain risk factors are predictive of post-ventriculostomy hemorrhage, the findings are relatively inconsistent throughout the literature [3–8]. In a retrospective study, Foreman et al. identified age greater than 50 years, pre-placement antithrombotic use, and an INR greater than 1.4 as being risk factors associated with the development of post-EVD hemorrhage [7]. However, the results of two previous studies failed to identify an elevated INR as high as 1.6 as being a statistically significant predictor of post-EVD ICH [5, 6]. The results of the single-center, retrospective study conducted by Sussman et al. suggest that age greater than 75 years is an independent predictor of catheter-related bleeding events; however, the results did not identify pre-placement antithrombotic use as a significant risk factor [5]. While advanced age was identified as a risk factor in two independent studies, a study by Maniker et al. suggests that age is not predictive of post-ventriculostomy hemorrhage [8].

In the present study, we assessed the impact of several different variables on the risk of hemorrhage after EVD insertion. While it has been suggested that advanced age, elevated INR, and hypertension may place patients at a higher risk for experiencing an post-EVD ICH, no statistically significant differences were found between the two groups of this study with respect to age, INR, and hypertension [5, 7]. Additionally, no difference was found between the two groups with respect to the number of passes with the ventricular catheter or the skill and experience of the operator. While controlling for potential confounders, the only variable found to be associated with post-EVD ICH was the postoperative use of an antiplatelet within 96 h of EVD insertion. Compared to those who did not receive an antiplatelet agent within 96 h after the placement of an EVD, those study participants who did receive postoperative antiplatelet therapy were 13.1 times (95% CI 1.95–88.6; *p* = 0.008) more likely to exhibit a new or enlarged ICH. This finding suggests that early postoperative use of an antiplatelet within 96 h of EVD insertion may significantly increase the risk of secondary hemorrhagic complications.

Although previous studies have failed to identify post-placement antiplatelet use as a significant risk factor for EVD-related hemorrhage, this association is intuitive. In a case-control study by Miller et al., decreased platelet levels on admission were associated with post-EVD hemorrhage [4]. Though thrombocytopenia and antiplatelet use are not the same, the Miller et al. study and our study suggest that decreased platelet activity in the perioperative period may be associated with a higher proportion of post-EVD hemorrhage. Consequently, it may be reasonable to avoid the routine use of antiplatelet therapy within 96 h after the placement of an EVD. However, antiplatelet agents should not be withheld due to concerns for hemorrhage when indicated for acute, life-threatening conditions.

Our study is not without limitations. As with all retrospective studies, we are limited by the availability of documented data. We excluded a significant number of patients due to incomplete documentation. However, our final logistic regression model had an appropriate fit based on the Hosmer and Lemeshow Goodness-of-Fit test (*p* = 0.5545). Because we were limited by sample size on the number of variables we could put in the logistic regression model, there may be other variables that contribute to the risk of new or enlarged hemorrhage that we were unable to measure. As this was a single center study, the results reported may not be generalizable to all patients undergoing the placement of an EVD. However, our results are consistent with other studies. This contributes to the generalizability of our results.

## Conclusion

In this study, the use of an antiplatelet within 96 h after the insertion of an EVD was associated with the development of post-EVD ICH. Based on the results from this study, the use of an antiplatelet within 96 h of EVD placement may significantly increase the risk of hemorrhagic complications. Therefore, when clinically appropriate, it may be reasonable to avoid antiplatelet use in the early postoperative time up to 96 h after insertion of an EVD.

## Abbreviations

CT: Computed tomography
EVD: External ventricular drain
GCS: Glasgow Coma Score
ICH: Intracranial hemorrhage
INR: International Normalized Ratio
VTE: Venous thromboembolism
